# Caffeic Acid in Spent Coffee Grounds as a Dual Inhibitor for MMP-9 and DPP-4 Enzymes

**DOI:** 10.3390/molecules28207182

**Published:** 2023-10-19

**Authors:** Enade P. Istyastono, Nunung Yuniarti, Vivitri D. Prasasty, Sudi Mungkasi, Stephanus S. W. Waskitha, Michael R. S. Yanuar, Florentinus D. O. Riswanto

**Affiliations:** 1 Research Group of Computer-Aided Drug Design and Discovery of Bioactive Natural Products, Faculty of Pharmacy, Sanata Dharma University, Yogyakarta 55282, Indonesia; s.waskitha@usd.ac.id (S.S.W.W.); gregoriusrest@gmail.com (M.R.S.Y.); dikaocta@usd.ac.id (F.D.O.R.); 2 Department of Pharmacology and Clinical Pharmacy, Faculty of Pharmacy, Universitas Gadjah Mada, Yogyakarta 55281, Indonesia; nunung@mail.ugm.ac.id; 3 Department of Pathobiological Sciences, School of Veterinary Medicine, Louisiana State University, Baton Rouge, LA 70803, USA; vprasasty@lsu.edu; 4 Department of Mathematics, Faculty of Science and Technology, Sanata Dharma University, Yogyakarta 55282, Indonesia; sudi@usd.ac.id

**Keywords:** type 2 diabetes mellitus, diabetic foot ulcers, caffeic acid, molecular dynamics simulations, PyPLIF HIPPOS 0.2.0

## Abstract

Type 2 diabetes mellitus and diabetic foot ulcers remain serious worldwide health problems. Caffeic acid is one of the natural products that has been experimentally proven to have diverse pharmacological properties. This study aimed to assess the inhibitory activity of caffeic acid and ethanolic extract of spent coffee grounds targeting DPP-4 and MMP-9 enzymes and evaluate the molecular interactions through 50-ns molecular dynamics simulations. This study also introduced our new version of PyPLIF HIPPOS, PyPLIF HIPPOS 0.2.0, which allowed us to identify protein–ligand interaction fingerprints and interaction hotspots resulting from molecular dynamics simulations. Our findings revealed that caffeic acid inhibited the DPP-4 and MMP-9 activity with an IC_50_ of 158.19 ± 11.30 µM and 88.99 ± 3.35 µM while ethanolic extract of spent coffee grounds exhibited an IC_50_ of 227.87 ± 23.80 µg/100 µL and 81.24 ± 6.46 µg/100 µL, respectively. Molecular dynamics simulations showed that caffeic acid interacted in the plausible allosteric sites of DPP-4 and in the active site of MMP-9. PyPLIF HIPPOS 0.2.0 identified amino acid residues interacting more than 10% throughout the simulation, which were Lys463 and Trp62 in the plausible allosteric site of DPP-4 and His226 in the active site of MMP-9.

## 1. Introduction

Type 2 diabetes mellitus (T2DM) is a metabolic disorder characterized by chronically increased blood glucose, insulin, and the dysfunction of pancreatic β-cells [1,2]. T2DM is also commonly associated with diabetic foot ulcers (DFU), which is a complication of severe and not well-controlled T2DM [3]. The International Diabetes Federation reported that about 537 million adults were living with diabetes in 2021, of whom more than 90% of people suffered from T2DM; this disease is estimated to rise in 2030 and 2045 [4]. Many treatments have been developed to cope with T2DM and DFU, including the utilization of natural products and their extracts [5,6,7]. As time goes by, natural products have played a pivotal role due to their ability to interact with various targets, leading to a significant contribution to drug discovery [8].

Caffeic acid, (*E*)-3-(3,4-dihydroxyphenyl)prop-2-enoic acid, is a phenolic compound that has widely been found in several agricultural products such as vegetables, fruits, carrots, tea, coffee beans, and spent coffee grounds [9,10]. Several experiments have documented that caffeic acid exhibited an extensive range of biological activities such as antioxidant [11], antiviral [12], anticancer [13], antiplasmodial [14], and antidiabetic properties [15,16]. Previous studies have found that caffeic acid has various diabetes-relieving mechanisms, including improving glucose homeostasis as well as pancreatic β-cell function [16], suppressing hepatic nuclear factor-4 (HNF-4), and decreasing phosphoenolpyruvate carboxykinase (PEPCK) activity to maintain glucose homeostasis [17]. Several natural products and their extracts targeting potential receptors for diabetes treatment such as dipeptidyl peptidase-IV (DPP-4) [18] and matrix metalloproteinase-9 (MMP-9) for wound healing of DFU have been well studied and reviewed [19,20,21,22]. Our previous in silico study also revealed the molecular mechanism of caffeic acid in inhibiting DPP-4 and that PyPLIF HIPPOS could identify the interaction hotspots [23,24]. Yet, the molecular mechanism and interaction hotspot identification of caffeic acid as a dual inhibitor targeting DPP-4 and MMP-9 for T2DM and DFU treatment are still rarely discussed.

This study aimed to experimentally evaluate the inhibitory activity of caffeic acid and ethanolic extract of spent coffee grounds targeting DPP-4 and MMP-9 enzymes. Furthermore, a molecular mechanism of caffeic acid as a dual inhibitor of DPP-4 and MMP-9 was investigated using 50-ns molecular dynamics simulations; its interaction hotspots were identified throughout the simulation. This research also introduced our new version of PyPLIF HIPPOS, PyPLIF HIPPOS 0.2.0., which is the upgraded version of PyPLIF HIPPOS, facilitated with a feature to identify interaction fingerprints directly from the protein–ligand complex in mol2, pdb, or pdbqt formats called ‘direct IFP’. This feature was applied to the complexes resulting from molecular dynamics simulations in this research.

## 2. Results and Discussion

### 2.1. DPP-4 and MMP-9 Inhibitory Assay

Spent coffee grounds are the solid waste by-products related to coffee consumption [25]. As waste products, spent coffee grounds are reported to have several organic compound contents such as polyphenols, amino acids, minerals, polysaccharides, and fatty acids [26]. Spent coffee grounds were also reported to have several benefits to human health due to their bioactive compound content [27]. In this study, we focused on the evaluation of the activity of spent coffee grounds towards both DPP-4 and MMP-9 inhibition. Caffeic acid standard activity was also evaluated in order to present the empirical results of bioactive compound activities. Furthermore, the molecular dynamics simulations were employed to strengthen the results of the in vitro evaluation. 

The DPP-4 inhibitory assay was performed using the DPP-4 inhibitor screening assay kit (Cayman Chemical, Ann Arbor, MI, USA). It was found that the half-maximal inhibitory concentration (IC_50_) of standard caffeic acid and ethanolic extract of spent coffee grounds toward DPP-4 were 158.19 ± 11.30 µM and 227.87 ± 23.80 µg/100 µL, respectively. The MMP-9 inhibitory assay was performed using the MMP-9 Inhibitor Screening Kit (Biovision K844-100). It was found that the IC_50_ of standard caffeic acid and the ethanolic extract of spent coffee grounds toward MMP-9 were 88.99 ± 3.35 µM and 81.24 ± 6.46 µg/100 µL, respectively. The lower IC_50_ value of both standard caffeic acid and ethanolic extract of spent coffee grounds towards MMP-9 compared to DPP-4 indicated the higher inhibitory activity of caffeic acid. 

Compared to other known DPP-4 inhibitors such as alogliptin, sitagliptin, and omarigliptin, caffeic acid tended to have a lower inhibitory activity [28,29]. Nevertheless, it was found that caffeic acid inhibited DPP-4 with a lower IC_50_ in µM compared to other natural products such as hopeaphenol [30], rutin, naringin, hesperidin, and eriocitrin [18,31]. Our results also exhibited that the ethanolic extract of spent coffee grounds had a lower IC_50_ in µg/µL compared to another natural product extract such as amla fruit extract from *Emblica officinalis* fruit [32]. Several MMP-9 inhibitors have been reviewed and exhibited a higher inhibitory activity compared to caffeic acid, such as marimastat together with its analogs, and sulfonamide hydroxamic acid-based inhibitors [33]. Interestingly, caffeic acid exhibited a lower IC_50_ value in µM compared to other natural products such as luteolin-7-*O*-rutinoside [34], kaempferol, morin, and taxifolin [35]. It was also discovered that the ethanolic extract of spent coffee grounds had a lower IC_50_ value in µg/mL than another natural product extract from the previous research, namely *Hibiscus rosa-sinensis* leaves [36]. Our results proved that caffeic acid and the ethanolic extract of spent coffee grounds could potentially be dual inhibitors of DPP-4 and MMP-9 as other natural product-based inhibitors for alternative T2DM and DFU treatment. To provide a comprehensive evaluation of the inhibitory effect, the molecular dynamics study was then applied to assess the interaction stability of caffeic acid towards DPP-4 and MMP-9.

### 2.2. Molecular Docking Simulations of Caffeic Acid Targeting DPP-4 and MMP-9

Redocking of DPP-4 and MMP-9 native ligands was initially performed prior to the molecular docking simulations of caffeic acid to validate the reliability of the used molecular docking configuration. Our results exhibited that 100 redocking simulations of the native ligand of each receptor had an RMSD value of ≤2.000 Å, suggesting that the redocking configuration was reliable and could be used for molecular docking of the proposed compound, i.e., caffeic acid in the active site of DPP-4 and MMP-9. 

The molecular docking simulations of caffeic acid in the active site of DPP-4 showed that all the best-docked caffeic acid had an RMSD value of ≤2.000 Å with a maximum value of 0.1183 Å, meaning that there was only one dominant pose of best-docked caffeic acid from 100 molecular docking replications. Compared to the molecular docking of caffeic acid in the DPP-4 active site, this investigation found that the best-docked caffeic acid poses in the MMP-9 active site resulted in RMSD value of ≤2.000 Å and >2.000 Å which asserted that there were more than one reasonable docking poses. In addition, it was discovered that the best-docked caffeic acid was stable enough to interact in the active site of DPP-4 with a binding affinity value of 6.707 to 6.798 kcal/mol while caffeic acid in the active site of MMP-9 also interacted nicely with a binding affinity value of 7.319 to 7.386 kcal/mol. To evaluate the interaction stability of each receptor–ligand complex, molecular dynamics simulations were performed as a further molecular docking validation since it was mentioned in the previous research [37]. In this research, the best-docked caffeic acid with the highest binding affinity was subjected to molecular dynamics simulations since it possessed the highest interaction stability.

### 2.3. Molecular Dynamics and Interaction Hotspot Identifications of Caffeic Acid-DPP-4

**Figure 1:**
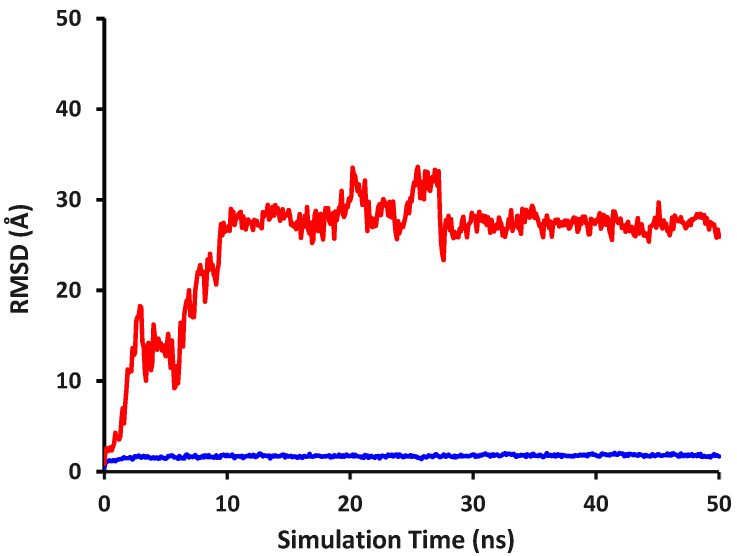
RMSD alterations of DPP-4 backbone atoms complexed with caffeic acid (blue) and RMSD ligand movement of caffeic acid (red) during 50-ns simulations.

The interaction stability of caffeic acid in the active site of DPP-4 and the conformational stability of DPP-4 backbone atoms were evaluated based on the RMSD ligand movement (RMSD_LigMove) and RMSD backbone, respectively, which are presented in Figure 1. According to the previous research [38], a ligand is considered to be stable if the RMSD value is below 2.000 Å. It can be clearly seen in Figure 1 that the RMSD LigMove values of caffeic acid never reached an RMSD value below 2.000 Å which indicated that caffeic acid interacted unstably in the DPP-4 active site, leading the caffeic acid explored in the DPP-4 allosteric sites to reach its maximum interaction stability [23]. The instability of caffeic acid interacting in the DPP-4 active site was presumably due to the negatively charged amino acid residues of Glu205 and Glu206 which electrostatically repelled the deprotonated caffeic acid. As reported by our previous research and Musoev et al., the active site of DPP-4 consisted of Tyr666, Tyr662, Tyr631, Tyr547, Phe357, Glu205, Glu206, and Arg125 [23,39]. Our visual inspection showed that caffeic acid still interacted with Tyr666, Tyr662, Tyr547, Phe357, Glu205, and Glu206 at the simulation time of 1.0 ns even though the RMSD LigMove value was 3.755 Å. The RMSD LigMove value increased until the simulation time reached 1.5 ns. At this time, caffeic acid interacted only with amino acid residues in the active site of Tyr547 and Phe357 with others of Pro550, Cys551, Ser552, Gln553, Trp629, and Ser630. 

From this simulation time, it could be seen that the RMSD LigMove value increased gradually along the simulation time until it reached 3.0 ns with an RMSD value of 18.158 Å while caffeic acid was no longer interacting with amino acid residues in the active side, revealing the availability of allosteric sites [40]. Even though there was a decreasing RMSD value reaching 9.209 Å at a simulation time of 5.7 ns, caffeic acid remained interacted in a plausible allosteric site. These results suggested that caffeic acid started escaping the active site at the simulation time of 1.5 ns to occupy more stable interactions in the plausible sites. Hence, caffeic acid might never interacte stably in the DPP-4 active site and might be a non-competitive inhibitor of DPP-4 as several non-competitive inhibitors, as previously reported [40,41,42]. 

Our findings showed that caffeic acid interacted with Trp62, Ser460, and Lys463 in the plausible allosteric site at the end of the simulation. It was found that caffeic acid formed ionic, hydrogen bond, and hydrophobic interactions with Lys463. The interactions were also strengthened by amino acid residues of Trp62 along with Ser460 via hydrogen bond with the carboxylic group of caffeic acid as the acceptor and amino acid residue of Trp62 via aromatic (edge-to-face together with face-to-face). These results revealed that the carboxylic group of caffeic acid played a significant role in receptor–ligand stabilization, forming an ionic interaction with Lys463 as well as hydrogen bonds with Trp62, Ser460, and Lys463. In addition, the aromatic ring of caffeic acid formed aromatic and hydrophobic interactions with Trp62 together with Lys463, respectively. 

Figure 1 also shows the alterating RMSD value of DPP-4 backbone atoms having an average RMSD value of 1.714 Å. Although there were seven snapshots having an RMSD value of more than 2.000 Å, the maximum value of the RMSD backbone was only 2.062 Å, hence it may be presumed that DPP-4 backbone atoms conformations were relatively stable during 50-ns simulations. Table 1 and Table 2 tabulate the amino acid residues interacting with caffeic acid for every snapshot which resulted from PyPLIF HIPPOS 0.2.0. Table 1 and Table 2 show that throughout the simulation, caffeic acid interacted with amino acid residues of Lys463 (47.50%) and Trp62 (12.57%) dominantly. Our investigations found that Lys463 forming an hydrophobic interaction and Trp62 forming an aromatic edge-to-face interaction were considered as the interaction hotspots located in the DPP-4 allosteric site which predominantly strengthened the caffeic acid interaction in the DPP-4 allosteric site.

### 2.4. Molecular Dynamics and Interaction Hotspot Identifications of Caffeic Acid-MMP-9

**Figure 2:**
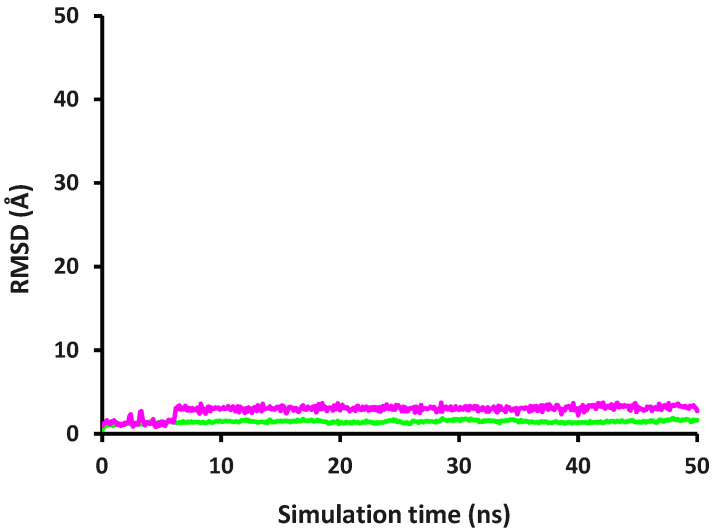
RMSD alterations of MMP-9 backbone atoms complexed with caffeic acid (green) and RMSD ligand movement of caffeic acid (purple) during 50-ns simulations.

Figure 2 illustrates the interaction stability of caffeic acid in the MMP-9 active site and the conformational stability of MMP-9 backbone atoms described with the alterations of the RMSD value. Our visual inspection exhibited that at the initial simulation time, caffeic acid interacted with amino acid residues of His226, Glu227, His236, and Pro246, which were reported as the catalytic domain of MMP-9 [43]. Unlike caffeic acid in the DPP-4 active site, caffeic acid comparatively stably interacted in the MMP-9 active site even though there were RMSD fluctuations reaching more than 2.000 Å.

It can be obviously observed in Figure 2 that caffeic acid interacted stably in the MMP-9 active site with an RMSD value below 2.000 Å until the simulation time reached 2.2 ns. From this simulation time, the RMSD value fluctuated until 6 ns with a maximum RMSD value of 2.708 Å. Starting from 6 ns to the end of the simulation, the RMSD value increased and continuously fluctuated, ranging from 1.373 to 3.729 Å. Even though it was found that the highest RMSD value was 3.729 Å at 42.1 ns, our findings suggested at that simulation time caffeic acid still interacted in the MMP-9 active site binding with amino acid residues of His226, Glu227, and His236. As expected, our results revealed that the carboxyl group of caffeic acid interacted with the Zn^2+^ ion throughout the simulation time via electrostatic interactions, denoting that caffeic acid has the same mechanism of MMP-9 inhibition as the other inhibitors previously reported [33]. Figure 2 also depicts the alterations of the RMSD value of MMP-9 backbone atoms having a value RMSD of ≤2.000 Å throughout the simulation, showing the stability of MMP-9 backbone atoms.

As tabulated in Table 3, PyPLIF HIPPOS 0.2.0 generated interaction hotspots of caffeic acid in the active site of MMP-9, revealing that His226 forming aromatic edge-to-face, aromatic face-to-face, and hydrophobic interactions were the interaction hotspots throughout the simulation. It was also observed that caffeic acid nicely interacted with Pro246 via hydrophobic interaction even though this amino acid had a minor contribution in stabilizing the interaction throughout the simulation. Our findings indicated that interaction with His226 had a major contribution to receptor–ligand stabilization, showing the importance of the aromatic group of caffeic acid in inhibiting MMP-9. 

According to the results of the molecular dynamics simulations (Table 1, Table 2 and Table 3), the feature of caffeic acid that is responsible for the inhibitory activity of both DPP-4 and MMP-9 was the presence of the aromatic moiety. This moiety interacts with Trp62 in the allosteric site of DPP-4 and His226 in the active site of MMP-9. Moreover, the presence of the carboxylic acid makes caffeic acid it possible to perform hydrogen bonds with either Trp62, Ser460, or Lys463 and an ionic interaction with Lys463 in the DPP-4 allosteric site. This carboxylic acid moiety could act as an electron-withdrawing group into the aromatic moiety of caffeic acid. This aromatic moiety could increase the aromatic interactions of caffeic acid to His226 of MMP-9. These features were also observed in the flavonoids that have inhibitory activity to DPP-4 and MMP-9 [44,45]. Hence, it is of considerable interest to take into account these findings in the design of dual active DPP-4 and MMP-9 inhibitors.

## 3. Materials and Methods

### 3.1. Chemicals

Caffeic acid (CAS No.: 331-39-5) was purchased from Sigma-Aldrich (St. Louis, MO, USA). Robusta coffee (Excelso^®^ Robusta Gold) was purchased from Kapal Api Grup, Jakarta, Indonesia. The DPP-4 inhibitor screening assay kit (Cayman Chemical, Ann Arbor, MI, USA) and MMP-9 inhibitor screening kit (Biovision K844-100) were used in this study. Dimethyl sulfoxide (DMSO), methanol, and ethyl acetate were also purchased from Sigma-Aldrich; distilled water was used as the solvent.

### 3.2. In Silico Instrumentations

A PC client having computer specifications of AMD Ryzen 7 4800H central processing unit, NVIDIA GeForce GTX 1650 Ti graphics processing unit, 16 GB of RAM, and 512 GB of SSD was used to perform all molecular docking simulations and identify receptor–ligand interaction hotspots resulting from molecular dynamics snapshots using PyPLIF HIPPOS 0.2.0. In addition, molecular dynamics simulations were conducted using a PC having specifications of AMD Ryzen 7 5800X central processing unit, NVIDIA GeForce GTX 1650 graphics processing unit, 32 GB of RAM, and 466 GB of SSD.

### 3.3. Standard Caffeic Acid and Sample Preparation

Preparation of standard caffeic acid and sample were developed according to the previous study [10]. Stock solutions of caffeic acid were prepared in methanol solvent. Then, 5 g of robusta coffee were accurately weighed and placed in a beaker glass followed by dilution using 200 mL of water (60 °C). This solution was then stirred four times followed by brewing for 5 min. The filtrate was separated and dried for 24 h at 60 °C. Three grams of spent coffee grounds was placed in a beaker glass and diluted with ethanol–water (40:60 *v*/*v*) for 2 h followed by stirring at 350 rpm and temperature of 60 °C. The obtained concentrate was then processed by applying liquid–liquid extraction using ethyl acetate (20 mL × 3). The achieved residue was diluted using methanol. 

### 3.4. DPP-4 Inhibitory Assay

The tested samples were dissolved and diluted using DMSO to the required concentration (500; 250; 125; 62.5; and 31.25 μM for caffeic acid and 500; 250; 100; 50; and 10 μg for ethanolic extract of spent coffee grounds in 100 μL assay final volume). The assay procedure is described briefly according to the manufacturer’s protocols as follows: diluted assay buffer (30 μL) and diluted enzyme solution (10 μL) were added to the 96-well plate containing 10 μL of solvent (negative control), sitagliptin (positive control), or test samples. As for the background well, the diluted enzyme solution was replaced by a diluted assay buffer. The reaction was initiated by adding 50 μL of a diluted substrate solution and the plate was incubated for 30 min at 37 °C. After incubation, fluorescence was read using a Synergy HTX-3 multimode reader at 350/450 nm. The percent inhibition was determined by the formula shown in Equation (1) and the IC_50_ can be calculated from linear regression log concentration vs percent inhibition.

(1) $$Inhibition\;(\%)=\left(\frac{Negative\;control-Inhibitor}{Negative\;control}\right)\times 100\%$$

### 3.5. MMP-9 Inhibitory Assay

A caffeic acid with a concentration of 250; 125; 62.5; and 31.25 μΜ and ethanolic extract of spent coffee grounds of 500; 250; 100; 50; and 10 μg in 100 μL assay final volume. The assay procedure is described briefly according to the manufacturer’s protocols as follows: the enzyme control was prepared by mixing 5 μL of the diluted MMP-9 with 45 μL buffer. The background control was prepared with 50 μL of buffer alone. Samples were prepared by mixing 1 μL sample with 5 μL diluted MMP-9 and 44 μL buffer while solvent control was prepared with the same method as the sample by adding 1 μL DMSO. The positive control was prepared by mixing 2 μL NNGH with 5 μL diluted MMP-9 and 43 μL buffer. The reaction was initiated by adding 50 μL of a diluted substrate solution. The fluorescence was read using a Synergy HTX-3 multimode reader at 325/393 nm in a kinetic mode at 37 °C for 60 min. Two time points were chosen in the linear range of enzyme kinetics to obtain the corresponding Δ relative fluorescence unit or RFU (RFU2-RFU1) during the reaction time Δt (at least 10 min apart). These values are used to obtain the percentage of inhibition which is formulated in Equation (2).

(2) $$Relative\;Inhibition\;(\%)=\frac{\Delta RFU\left(ec\right)-\Delta RFU(sample)}{\Delta RFU(ec)}\times 100\%$$

### 3.6. Receptor–Ligand Preparation

The crystal structure of the receptors, namely DPP-4 complexed with its native ligand, i.e., alogliptin (PDB ID: 3G0B) and MMP-9 complexed with its native ligand, i.e., CC27 (PDB ID: 4H3X), were downloaded directly to the YASARA-structure. The DPP-4 receptor was prepared by omitting unimportant residues (Nag) and solvent molecules (Hoh) while chain A was selected for further simulations. Subsequently, the pH value was adjusted to physiological pH with the commands of *pH value = 7.4*, *update = yes*, and *CleanAll* to add hydrogen atoms correctly at the determined pH. The energy minimization was performed using commands of *Options > Choose Experiment > Energy minimization*. This prepared structure, DPP-4 complexed with alogliptin, was saved as 3G0B-corr-min.yob in the working directory. 

The MMP-9 receptor was prepared by omitting its solvent molecules (Hoh) and unimportant residues (Peg, Pgo, Gol, Ca^2+^, and Zn^2+^ which are not complexed with the native ligand) while chain A was selected for further simulations. In this preparation, we discovered that YASARA cannot identify the single bond of the NH-OH group of the native ligand. Therefore, we manually corrected it by adjusting the system pH to physiological pH with a command of *pH value = 7.4*, *update = yes*, and *CleanAll* and editing that bond from double bond to single bond with a command of DelBond 2669,2668 followed by a command of AddBond 2669, 2668 and AddHyd all using the YASARA-console. Finally, energy minimization was also performed using the same commands as DPP-4 preparation and the Zn^2+^ object was joined to the receptor, becoming one object containing the receptor and the Zn^2+^. The prepared structure of MMP-9 complexed with CC27 was saved as 4H3X-corr-min.yob in a different working directory.

### 3.7. Redocking of the Native Ligand

Redocking of the native ligand was carried out to validate the proposed molecular docking configuration [46]. Redocking of each native ligand for each receptor was conducted using our in-house developed YASARA plug-in which enabled us to perform 100 redocking simulations. The prepared and corrected receptor–ligand complex file, 3G0B-corr-min.yob and 4H3X-corr-min.yob, was individually set as the MacroTarget for our in-house developed YASARA plug-in. The processes involved in our in-house developed YASARA plug-in are: (1) the receptor–ligand complex was separated into two objects which were object 1 as the receptor and object 2 as the native ligand, respectively. At the same time, this process also constructed object 3, which was a cubic simulation cell with a distance of 5.000 Å from the native ligand outer atoms. (2) Afterward, object 2 was saved individually as a YASARA object file with the file name of MacroTarget_ref.yob and MacroTarget_ligand.yob then object 2 was deleted from the YASARA-structure. (3) Another file was generated, i.e., MacroTarget_receptor.sce, containing the receptor structure and the simulation cell. Subsequently, 100 redocking simulations of MacroTarget_ligand.yob and MacroTarget_receptor.sce were performed using the AutoDock Vina scoring function [47]. Eventually, the root-mean-square deviation (RMSD) of the best-docked native ligand from each simulation was calculated, comparing the heavy atom coordinate and native ligand conformation to the co-crystalized native ligand structure as saved as MacroTarget_ref.yob. Our in-house developed YASARA plug-in also generated a file named MacroTarget_config.mcr containing the redocking configuration, which could be used for the molecular docking configuration of the proposed compound, i.e., caffeic acid, with the same configuration as the redocking.

### 3.8. Molecular Docking of Caffeic Acid

Molecular docking of caffeic acid targeting the DPP-4 and MMP-9 active sites was conducted separately in a different working directory. MacroTarget_receptor.sce and MacroTarget_config.mcr resulted from the redocking of each native ligand and were copied as the files generated from each redocking process. The three-dimensional structure of caffeic acid was built using the YASARA-structure and its SMILES code of C1=CC(=C(C=C1C=CC(=O)O)O)O. The pH system was also adjusted to physiological pH with a command of *pH value = 7.4*, *update = yes*, and *CleanAll* and then to add hydrogen atoms in that desired pH condition. The energy minimization was performed using commands of *Options > Choose Experiment > Energy minimization*. This 3D molecular model of caffeic acid was then saved as 3G0B-corr-min.yob and 4H3X-corr-min.yob as the prepared molecular model to perform molecular docking in the DPP-4 and MMP-9 active sites, respectively. Each file was set as the MacroTarget for our other in-house developed YASARA plug-in on YASARA-structure, allowing us to set the molecular docking configuration as the redocking configuration which was set in MacroTarget_config.mcr file. Thereafter, our subsequent in-house developed YASARA plug-in was run to perform 100 molecular docking simulations. At last, the RMSD value of the best-docked caffeic acid was calculated, comparing the heavy atom coordinate and conformation of the best-docked caffeic acid from each simulation with the best-docked caffeic acid from the first simulation. This RMSD calculation was run using our next in-house developed YASARA plug-in resulting in a file named rmsd_bestpose_all.txt. The best-docked caffeic acid which possessed an RMSD value > 2.000 Å denoted that the best-docked caffeic acid conformation could be distinguishable from the best-docked caffeic acid resulted from the first simulation.

### 3.9. Molecular Dynamics Simulations

The interaction stability of the best-docked caffeic acid in the active site of DPP-4 and MMP-9 was evaluated using molecular dynamics simulations which were executed using the YASARA-structure. Molecular dynamics simulations were performed using the AMBER14 force field. The solvation system was set in the 10 × 10 × 10 Å^3^ cubic cell and a periodic boundary condition (PBC) was maintained during the simulations with water density of 0.993 g/mL at 310 K in the pressure of 1 bar. The system was neutralized by the addition of counter ions, Na^+^ and Cl^−^, with a concentration of 0.9% and the pH was set to 7.4 as the physiological condition. The system was then subjected to energy minimization using the steepest descent and simulated annealing to remove steric clashes. The particle mesh Ewald (PME) algorithm with no cut-off was used to describe the long-range electrostatic interactions whereas the van der Waals interactions cut-off was 8.00 Å. Finally, molecular dynamics simulations of each system were carried out in 50-ns with a time step of 2.5-fs and the snapshot was saved every 100-ps. The molecular dynamics simulations resulted in root-mean-square deviation of receptor backbone atoms (RMSD-backbone) and root-mean-square deviation of ligand movement (RMSD-LigMove) which were then further analyzed to evaluate the stability of receptor–ligand interactions. 

### 3.10. Interaction Hotspot Identifications

The identification of interaction fingerprints was carried out using PyPLIF HIPPOS 0.2.0., which is the new version of PyPLIF HIPPOS [24]. The snapshots resulting from the molecular dynamics simulations (see Section 3.9) were converted into pdb format. Subsequently, the molecular dynamics snapshots in pdb format were analyzed automatically using the new feature direct IFP from PyPLIF HIPPOS 0.2.0 to identify interaction fingerprints of caffeic acid interacting with DPP-4 and MMP-9 throughout the simulations. The resulting interaction fingerprints were grouped based on the interaction type to calculate the interaction hotspots.

## 4. Conclusions

Caffeic acid and the ethanolic extract of spent coffee grounds could potentially act as dual inhibitors targeting DPP-4 and MMP-9 as alternative treatments for type 2 diabetes mellitus and diabetic foot ulcers. Molecular dynamics simulations denoted that caffeic acid interacted in the plausible allosteric site of DPP-4 and the active site of MMP-9, which could support the in vitro experimental data. PyPLIF HIPPOS 0.2.0 successfully identified the interaction hotspots of caffeic acid interacting with DPP-4 and MMP-9 throughout 50-ns molecular dynamics simulations, which exhibited the predominant interactions reinforcing the receptor–ligand interactions. Our findings also provide the new version of PyPLIF HIPPOS, i.e., PyPLIF HIPPOS 0.2.0 which can be further used to support structure-based drug discovery.

## Figures and Tables

**Table 1:** Hydrophobic interaction hotspots resulted from 50-ns molecular dynamics simulations of DPP-4 interacting with caffeic acid.

Interacting Residue	Interaction Percentage
Arg61	0.80%
Trp62	1.80%
Pro159	2.40%
Pro218	1.60%
Lys463	47.50%
Tyr547	2.79%
Asn562	0.80%
Tyr631	0.60%
Tyr662	0.20%
Tyr666	1.40%

**Table 2:** Non-hydrophobic interaction hotspots resulted from 50-ns molecular dynamics simulations of DPP-4 interacting with caffeic acid.

Interacting Residue	Interaction Type	Interaction Percentage
Tyr48	Aromatic (Edge to face)	0.20%
Ser59	H-bond (Acceptor)	0.20%
Arg61	H-bond (Donor)	3.59%
Arg61	Ionic (Cation)	4.39%
Trp62	Aromatic (Edge to face)	12.57%
Trp62	Aromatic (Face to face)	1.00%
Trp62	H-bond (Donor)	0.20%
Ser106	H-bond (Donor)	1.60%
Glu206	H-bond (Acceptor)	0.80%
Phe357	Aromatic (Edge to face)	0.60%
Phe357	Aromatic (Face to face)	2.00%
Ser460	H-bond (Acceptor)	0.40%
Lys463	H-bond (Donor)	6.59%
Lys463	Ionic (Cation)	5.59%
Glu464	H-bond (Acceptor)	1.60%
Arg471	H-bond (Donor)	2.20%
Arg471	Ionic (Cation)	2.79%
Ser473	H-bond (Acceptor)	0.20%
Tyr547	Aromatic (Face to face)	0.60%
Lys554	H-bond (Donor)	0.80%
Lys554	Ionic (Cation)	1.20%
Arg560	H-bond (Donor)	1.20%
Arg560	Ionic (Cation)	1.40%
Asn562	H-bond (Donor)	1.20%
Tyr666	Aromatic (Edge to face)	1.60%
Tyr666	Aromatic (Face to face)	0.40%

**Table 3:** Interaction hotspots resulted from 50-ns molecular dynamics simulations of caffeic acid in the active site of MMP-9.

Interacting Residues	Interaction Type	Interaction Percentage
His226	Aromatic (Edge-to-face)	15.56%
	Aromatic (Face-to-face)	73.85%
	Hydrophobic	90.01%
	H-bond (Donor)	1.79%
Pro246	Hydrophobic	0.79%

## Data Availability

The data presented in this study are available on request from the corresponding author.

## Sample Availability

Samples of the compounds are available from the authors.
